# Intravitreal Injection of Conbercept Combined with Minimally Invasive Photocoagulation and Phacoemulsification Intraocular Lens Implantation in the Treatment of Neovascular Glaucoma Complicated with Cataract

**DOI:** 10.1155/2023/2834398

**Published:** 2023-02-10

**Authors:** Xue Li, Dawei Zhang

**Affiliations:** Beijing Luhe Hospital Affiliated to Capital Medical University of Ophthalmology, No. 82 Xinhua South Road, Tongzhou District, Beijing, China

## Abstract

**Background:**

Neovascular glaucoma (NVG) is a complex refractory glaucoma with a high rate of blindness. Compaq is an anti-VEGF agent that has been used in clinical NVG therapy.

**Objective:**

This study aims to investigate the effect of intravitreal injection of conbercept combined with minimally invasive photocoagulation and phacoemulsification intraocular lens implantation in the treatment of NVG complicated with cataracts.

**Methods:**

A total of 84 patients with NVG complicated with cataracts who were admitted to our hospital from September 2019 to September 2021 were selected. According to the random number table method, they were divided into the study group and the control group, with 42 cases in each. The control group underwent minimally invasive photocoagulation combined with phacoemulsification and intraocular lens implantation. The study group was given an intravitreal injection of conbercept first, followed by minimally invasive photocoagulation combined with phacoemulsification and intraocular lens implantation 3 to 7 days after the injection. The intraocular pressure and visual acuity of the two groups before surgery, 1 week, 1 month, 3 months, and 6 months after the operation were compared. The levels of serum vascular endothelial growth factor (VEGF) and interleukin-6 (IL-6) were compared between the two groups before the operation and 1 week after the operation. The incidence of postoperative complications in the two groups was statistically compared.

**Results:**

At 1 week, 1 month, 3 months, and 6 months after the operation, the intraocular pressure of the study group was lower than that of the control group, and the visual acuity was better than that of the control group (*P* < 0.05); one week after the operation, the serum levels of VEGF and IL-6 in the study group were lower than those in the control group (*P* < 0.05); there was no significant difference in the incidence of complications between the two groups (*P* > 0.05).

**Conclusions:**

Intravitreal injection of conbercept combined with minimally invasive photocoagulation and phacoemulsification intraocular lens implantation in the treatment of NVG complicated with cataract can improve the levels of serum inflammatory factors and vasoactive substances, effectively reduce intraocular pressure, and improve visual acuity.

## 1. Introduction

Neovascular glaucoma (NVG) is a complex refractory glaucoma with a high rate of blindness. The disease mostly occurs in people over 40 years of age, so timely and reliable measures should be given to avoid vision loss. The main causes are ocular ischemic syndrome, fundus retinal vein occlusion, diabetic retinopathy, and other ocular ischemic diseases. Clinical manifestations are difficult to control high intraocular pressure, irreversible visual impairment, and intractable eye pain, often complicated by a variety of eye diseases, such as cataracts, corneal degeneration, and so on [1, 2]. At present, for NVG patients with cataracts, trabeculectomy, goniosynechialysis combined with phacoemulsification, and intraocular lens implantation are mainly performed to reduce intraocular pressure and restore vision. However, the traditional antiglaucoma method is not ideal for various reasons. In recent years, it has been found that minimally invasive photocoagulation combined with cataract surgery can destroy the unpigmented epithelial cells of the ciliary process by laser, which can accurately control energy, improve filtration function, avoid the problem of insufficient or excessive filtration in traditional antiglaucoma surgery, and has high safety can better maintain postoperative vision [3, 4]. Vascular endothelial growth factor (VEGF) is a cytokine formed when tissues throughout the body are hypoxic and have the effect of promoting angiogenesis. The number of retinal neovascularization is closely related to the level of VEGF. In addition, patients with neovascular glaucoma have abnormally elevated levels of VEGF in aqueous humor. In other words, patients with neovascular glaucoma can secrete VEGF-A from the retinal pigment epithelial cells due to retinal hypoxia, thereby promoting choroidal neovascularization. At present, it has been confirmed that VEGF is the key link to intraocular neovascularization. Compaq, as an anti-VEGF drug, has been used in clinical NVG treatment [5, 6]. This study investigated the effect of intravitreal injection of conbercept combined with minimally invasive photocoagulation and phacoemulsification, and intraocular lens implantation in the treatment of NVG patients with cataracts. The report is as follows.

## 2. Material and Methods

### 2.1. General Information

Eighty-four cases of NVG with cataracts treated in our hospital from September 2019 to September 2021 were selected. Inclusion criteria were as follows: (1) all of them met the diagnostic criteria of NVG complicated with cataract by ophthalmological examination [7]; (2) intraocular pressure cannot be effectively controlled after treatment with 2 or more intraocular pressure drugs; (3) age ≥18 years old; (4) monocular lesions; (5) lens nucleus ≥ grade III. Exclusion criteria were as follows: (1) retinal detachment; (2) primary glaucoma; (3) patients with severe heart, liver, kidney, and lung dysfunction; (4) history of previous ophthalmic surgery; (5) had been treated with anti-VEGF drugs; (6) combined with other eye diseases; (7) early stage of cataract; (8) combined with the pronounced vitreous volume of blood; (9) patients with mental disorders; (10) patients with incomplete clinical data.

According to the method of the random number table, they were divided into a study group (*n* = 42) and a control group (*n* = 42). In the control group, there were 23 males and 19 females, aged 43–68 years old, with an average age of (55.36 ± 4.12), 26 cases of central retinal vein obstruction, and 16 cases of diabetic retinopathy. In the study group, there were 25 males and 17 females, aged from 45 to 69 years old, with an average of (56.23 ± 4.36) years. There were 27 cases of central retinal vein occlusion and 15 cases of diabetic retinopathy. The baseline data of the two groups were balanced and comparable (*P* > 0.05).

### 2.2. Method

The control group underwent minimally invasive photocoagulation combined with phacoemulsification and intraocular lens implantation: mydriasis, towel disinfection, eye opener eyelid, surgical eye surface anesthesia, the first phacoemulsification and intraocular lens implantation, 11 o 'clock position for the transparent corneal incision (2.2 mm), at 2 o 'clock position for auxiliary incision, the viscoelastic agent was injected into the anterior chamber, continuous circular capsulorhexis (diameter 5.5 mm) was performed by capsulorhexis forceps, water separation, phacoemulsification, aspiration of lens cortex, posterior capsule to maintain transparency, and integrity, a viscoelastic agent from the capsule bag, anterior chamber injection, and an intraocular lens from transparent corneal incision from the capsule bag injection. After completion of minimally invasive photocoagulation, viscoelastic agent ciliary groove deep injection, endoscopic probe through the transparent main incision into, through the pupil to the contralateral iris posterior, ciliary body area between the ciliary body sac, adjust the focal length, 150°–180° ciliary protrusion continuous photocoagulation, the number of points 18–35 times, energy 0.25–0.40 W, 0.2–0.6 s exposure time, real-time adjustment of parameters, the endoscopic monitor to see the ciliary body whitening, fold collapse, and no ciliary process reaction, as successful photocoagulation, after sucking the viscoelastic agent, equilibrium liquid from the auxiliary incision injection, water seal corneal incision, restore intraocular pressure. After the operation, tobramycin-dexamethasone eye drops were given for 4 weeks, combined with the local application of nonsteroidal eye drops for 8 weeks for anti-infection and anti-inflammation.

In the study group, conbercept was injected into the vitreous cavity first. The methods were as follows: mydriasis, towel disinfection, topical anesthesia of the eye, eyelid opening with an eye opener, and vertical puncture of the sclera through the pars plana of the ciliary body at 3.5–4.0 mm posterior to the infratemporal limbus to enter the vitreous cavity. 0.05 ml of conbercept at a concentration of 10 mg/ml (S20130012, Chengdu Kanghong Biotechnology Co., Ltd.) was injected, the needle was pulled out, the eyeball was locally compressed for 2 minutes, and ofloxacin eye ointment was applied. According to the regression of neovascularization and the inflammatory reaction of the anterior chamber, minimally invasive photocoagulation combined with phacoemulsification and intraocular lens implantation was performed 3–7 days after injection. The method of minimally invasive photocoagulation combined with phacoemulsification and intraocular lens implantation was the same as that of the control group. This study was approved by the hospital ethics committee. All patients sign an informed consent form. There were no patients who dropped out of the study.

### 2.3. Observation Index

(1) Intraocular pressure (IOP): IOP was measured before the operation, 1 week, 1 month, 3 months, and 6 months after the operation. (2) Visual acuity: best corrected visual acuity (BCVA) was measured using an international standard visual acuity chart before surgery, 1 week, 1 month, 3 months, and 6 months after surgery. (3) Serum VEGF and interleukin-6, IL-6 levels: fasting venous blood 3 ml was taken before the operation and 1 week after the operation. Serum VEGF (JL18341, Shanghai Jianglai Biotechnology Co., Ltd) and IL-6 (JL14113, Shanghai Jianglai Biotechnology Co., Ltd) were detected by enzyme-linked immunosorbent assay (JL18341, Shanghai Jianglai Biotechnology Co., Ltd). (4) The incidence of postoperative complications in the two groups was calculated.

### 2.4. Statistical Method

SPSS22.0 was used to analyze the data. Mean ± standard deviation (±*s*) was used to represent the measurement data, *t*-test or repeated measurement analysis of variance, *n* (%) was used to represent the count data, and *χ*2 test was used. *P* < 0.05 indicated that the difference was statistically significant.

## 3. Results

### 3.1. Intraocular Pressure

The results of repeated measures analysis of variance showed that the intraocular pressure of the two groups was statistically significant in terms of time, group, and interaction. (*P* < 0.05); at 1 week, 1 month, 3 months, and 6 months after the operation, the intraocular pressure in the study group was lower than that in the control group (*P* < 0.05). See Table 1.

### 3.2. Eyesight

The results of repeated measurement analysis of variance showed that the eyesight level of the two groups was statistically significant in terms of time, intergroup, and interaction (*P* < 0.05); the eyesight of the study group was better than that of the control group at 1 week, 1 month, 3 months, and 6 months after the operation. *P* < 0.05). See Table 2.

### 3.3. Serum VEGF, IL-6 Levels

One week after the operation, the levels of serum VEGF and IL-6 in the study group were lower than those in the control group (*P* < 0.05). See Table 3.

### 3.4. Complication

There was no significant difference in the incidence of complications between the two groups (*P* < 0.05), see Table 4.

## 4. Discussion

The pathogenesis of NVG is complex and is related to retinal ischemic disease. When the retina has hypoxia-ischemia, the eye VEGF will be oversecreted and circulate to the chamber angle and anterior chamber through the aqueous humor, resulting in the formation of neovascularization and a fibrous membrane, which will further pull the chamber angle and close its adhesion, resulting in the increase of intraocular pressure (IOP) [8, 9]. High intraocular pressure will aggravate the degree of retinal ischemia, form a vicious circle, and damage vision. Therefore, the key to NVG treatment is to inhibit neovascularization and reduce intraocular pressure.

Minimally invasive photocoagulation is an important method for the treatment of refractory glaucoma. It is performed under endoscopic direct vision, with accurate ciliary body positioning and accurate photocoagulation range, by reducing the formation of the aqueous humor photocoagulation ciliary body, to reduce the intraocular pressure effect, and can significantly reduce the surrounding tissue damage, reduce the risk of visual impairment of traditional photocoagulation [10]. Fu et al. [11] found that minimally invasive photocoagulation is used to treat refractory glaucoma, which can significantly reduce intraocular pressure in patients, have fewer complications, and can obtain similar effects as trabecular resection. NVG is often due to the iris surface and anterior chamber angle being covered with neovascularization, and simple surgical treatment is more prone to bleeding. Studies have shown that VEGF overexpression is one of the main mechanisms to induce angiogenesis [12], Therefore, the preoperative use of anti-VEGF drugs can promote the regression of neovascularization and inhibit its formation. Yu et al [13] also found that vitreous injection of anti-VEGF drugs before routine surgery can promote the regression of neovascularization, reduce the rate of intraoperative and postoperative bleeding, and ensure the smooth implementation of the operation. As a new recombinant fusion protein independently developed in China, the core region of conbercept is a fully humanized amino acid sequence with high affinity. Compared with natural receptors, monoclonal antibodies, and VEGF, conbercept is more closely combined, which can completely penetrate the retina and inhibit VEGF-induced angiogenesis [14–16]. Therefore, in this study, patients with NVG complicated with cataracts were treated with intravitreal injection of Combuxil, followed by minimally invasive photocoagulation combined with phacoemulsification and intraocular lens implantation 3–7 days after injection. The incidence of hyphema was low, but there was no significant difference between the two groups, which may be related to the small sample size of this study. There were no significant differences in the incidence of other complications, indicating a high safety profile.

The results of this study showed that the IOP of the study group was lower than that of the control group at 1 week, 1 month, 3 months, and 6 months after the operation. The reason is that with the help of the anti-VEGF drug Compaq, IOP can be quickly and effectively controlled within the normal range after NVG. The visual acuity of 6 months after the operation was better than that of the control group, indicating that intravitreal injection of the conbercept combined with minimally invasive photocoagulation, and phacoemulsification and intraocular lens implantation in the treatment of NVG with cataract has a significant curative effect in reducing intraocular pressure and improving visual acuity. IL-6 can mediate the body's inflammatory response and can also regulate VEGF expression in vivo. Related studies have shown that serum VEGF levels are positively correlated with aqueous humor levels [17]. This study found that the serum VEGF and IL-6 levels decreased one week after surgery, and the study group was lower than the control group, which confirmed that the use of Compaxipr can effectively reduce the production of inflammatory factors and vasoactive substances before minimally invasive photocoagulation combined with ultrasonic emulsifying intraocular lens implantation. This is mainly due to the fact that intravitreal injection of Compaq can combine with some VEGF-B and all VEGF-A, and then block neovascularization; after treatment, intraocular hypertension returns to normal, the intraocular microenvironment returns to a stable state, and the level of inflammatory factors decreases. There are also some shortcomings in this study, as the patients in this study are all from the same hospital and are not representative of the overall situation, which may lead to some bias in the results of this study. Moreover, this study has a short observation time and a small sample size, so it is necessary to further prolong the follow-up time and conduct a large sample study.

## 5. Conclusion

Vitreous injection of Compaq combined with minimally invasive photocoagulation and phacoemulsification intraocular lens implantation in the treatment of NVG complicated with cataract can improve the levels of serum inflammatory factors and vasoactive substances, effectively reduce intraocular pressure, and improve visual acuity.

## Figures and Tables

**Table 1:** Comparison of intraocular pressure at different time points in two groups (mmHg, $\bar{x}$ ±*s*).

Group	Study group	Control group	*F* time value/*P* value	*F* group value/*P* value	*F* interaction value/*P* value
Case	42	42			

Before surgery	41.32 ± 5.67	42.15 ± 6.32	345.267/<0.001	32.329/<0.001	16.158/<0.001
One week after surgery	20.25 ± 3.24^ab^	22.78 ± 3.45^a^			
One month after surgery	18.78 ± 3.01^ab^	20.45 ± 3.21^a^			
Three months after the operation	18.43 ± 3.15^ab^	19.97 ± 3.04^a^			
Six months after the operation	17.45 ± 3.23^ab^	19.55 ± 3.03^a^			

Compared with the same group before the operation, ^a^*P* < 0.05; compared with the control group at the same time, ^b^*P* < 0.05. The *F* value indicates the significance of the entire fitting equation, and the larger the *F*, the more significant the equation and the better the fit.

**Table 2:** Comparison of logMAR BCVA between two groups at different time points ($\bar{x}$ ±*s*).

Group	Study group	Control group	*F* time value/*P* value	*F* group value/*P* value	*F* interaction value/*P* value
Case	42	42			

Before surgery	1.21 ± 0.35	1.22 ± 0.36	176.486/<0.001	28.749/<0.001	17.324/<0.001
One week after surgery	0.85 ± 0.16^ab^	0.94 ± 0.17^a^			
One month after surgery	0.81 ± 0.18^ab^	0.92 ± 0.19^a^			
Three months after the operation	0.73 ± 0.17^ab^	0.85 ± 0.16^a^			
Six months after the operation	0.68 ± 0.15^ab^	0.80 ± 0.17^a^			

Compared with the same group before the operation, ^a^*P* < 0.05; compared with the control group at the same time, ^b^*P* < 0.05.

**Table 3:** Serum VEGF and IL-6 levels were compared in two groups (pg/mL, $\bar{x}$ ±*s*).

Group		Study group	Control group	*T* value	*P* value
Case		42	42		

VEGF	Before surgery	535.23 ± 72.47	541.39 ± 74.96	0.383	0.703
	1 week after surgery	356.72 ± 52.49^a^	416.38 ± 53.42^a^	5.163	<0.001

IL-6	Before surgery	273.81 ± 32.67	279.52 ± 33.19	0.795	0.429
	1 week after surgery	165.93 ± 26.74^a^	201.43 ± 28.18^a^	5.922	<0.001

Comparison with the same group before the operation, ^a^*P* < 0.05.

**Table 4:** Comparison of complications between the two groups *n* (%).

Group	Study group	Control group	*χ * ^*2*^ value	*P* value
Case	42	42		
Bleeding in the anterior chamber	1 (2.38)	3 (7.14)	1.053	0.306
Low intraocular pressure	1 (2.38)	2 (4.76)	0.346	0.557
Shallow anterior chamber	1 (2.38)	2 (4.76)	0.346	0.557
Endophthalmitis	1 (2.38)	3 (7.14)	1.053	0.306
Anterior chamber fibers oozate	2 (4.76)	3 (7.14)	0.213	0.645

## Data Availability

The datasets used and analyzed during the current study are available from the corresponding author upon reasonable request.
